# Wide Bandwidth Angle- and Polarization-Insensitive Symmetric Metamaterial Absorber for X and Ku Band Applications

**DOI:** 10.1038/s41598-020-67262-5

**Published:** 2020-06-25

**Authors:** Saif Hannan, Mohammad Tariqul Islam, Ali F. Almutairi, Mohammad Rashed Iqbal Faruque

**Affiliations:** 1 Department of Electrical, Electronic and Systems Engineering, Faculty of Engineering and Built Environment, Universiti Kebangsaan Malaysia, Bangi, 43600 Selangor Malaysia; 2 Electrical Engineering Department, College of Engineering and Petroleum, Kuwait University, Safat, 13060 Kuwait; 3 Space Science Center (ANGKASA), Universiti Kebangsaan Malaysia, 43600 UKM Bangi, Selangor, Malaysia

**Keywords:** Engineering, Physics

## Abstract

In this paper, a wide bandwidth angle- and polarization-insensitive symmetric metamaterial (MM) absorber for X and Ku band is proposed. For both normal and oblique incidence in TEM mode, the proposed unit cell shows high absorption at different polarizing angles due to structural symmetry. A four-fold resonator was introduced in the unit cell to enhance the bandwidth. The performance of the proposed absorber is determined by both full-wave simulations and measurements. The simulated and measured absorptions are almost similar at normal incidence with 94.63%, 95.58%, 97% and 75.58% at 11.31 GHz, 14.11 GHz, 14.23 GHz, and 17.79 GHz respectively. At 45° for these frequencies, the absorptions are 95.47%, 97.2%, 97.12% and 75.29% respectively. For 90°, the absorptions are similar to those for 45° except 98.15% for 14.21 GHz. At all these angles and resonance frequencies, either permittivity or permeability was found negative, as a result, the refractive index was negative revealing metamaterial characteristics of the unit cell. Along with high absorptivity and wide incidence angle insensitivity up to 90°, a total of 1.42 GHz of absorption bandwidth was achieved, which is better than recent similar works with FR4 substrate.

## Introduction

Metamaterial (MM) absorbers are recent developments in the field of electromagnetic wave applications like 5 G antenna, Radar cross-section reduction, remote sensing, stealth technology and photo-electron absorption in THz range, etc. MM absorbers are those materials that usually exhibit either negative permittivity or negative permeability or both are negative when electromagnetic (EM) waves pass through them^1–3^. As a result, they absorb most of the EM waves, as the transmission coefficient of MM absorber is negligible, and the reflection coefficient is very small. These properties are not present in any material generally used for EM wave applications unless they are engineered^4,5^. Research is going on to achieve absorbance of certain selective range or entire incident EM waves for purpose-wise applications^6–10^. Different types of substrate materials are used for appropriate di-electric properties for the desired absorbance of the EM waves^11^. FR4 (fire retardant 4) is one of the most popular and widely used substrates for MM absorber design^12^. Although FR4 is not appropriate because of high dielectric loss^13^ at high-frequency range like X and Ku band, it is popular because of low cost, availability and most importantly, absorber applications due to high dielectric loss.

The absorption capability of an MM absorber depends not only on the unit cell design but also on the angle of incidence of the incident EM wave and their polarization types. EM waves are sometimes needed to be absorbed for sensing devices or frequency-selective antenna where the type of polarization and incident angle is important for the absorption capacity of the absorber.

Research is going on to design MM absorbers with features like angle and polarization insensitiveness. The unit cell should also be symmetrically shaped like a split-ring cross resonator, circular sector, Jerusalem cross-section, four-fold symmetric and so on^14–19^. These absorbers can absorb EM waves with high efficiency at some certain frequencies with very low bandwidth. Hence, they are not appropriate for mass deployment into places for wide bandwidth absorption like radar cross-section reduction, remote sensing, unwanted frequency absorption in antennae, stealth capability in modern military airplanes and so on. To overcome all these challenges in this modern age, the MM absorbers should be designed with such precision that, they will be polarization and wide-angle insensitive, symmetric in structural design, capable of high absorption and most importantly, they must have broad bandwidth of absorption efficiency. For both X and Ku band, only a few absorbers are designed with FR4 to date. The efficiency of those absorbers is not at a satisfactory level concerning the afore-mentioned performance indicators.

In this paper, we propose an MM absorber which satisfies all these properties for X and Ku band applications. We also have compared the performance of the unit cell with recently published relevant absorbers and found it better in all aspects of the above-mentioned performance categories. The most popular FR4 substrate is used as the dielectric barrier for the unit cell, where the copper patch is backed up by a copper ground with the substrate in between them.

### Design of unit cell

**Figure 1:**
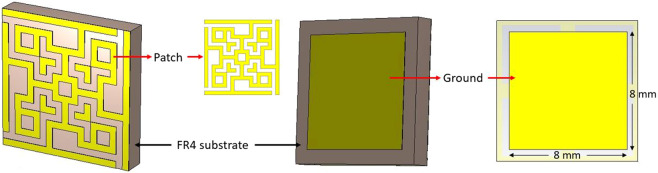
Design of the unit cell.

**Figure 2:**
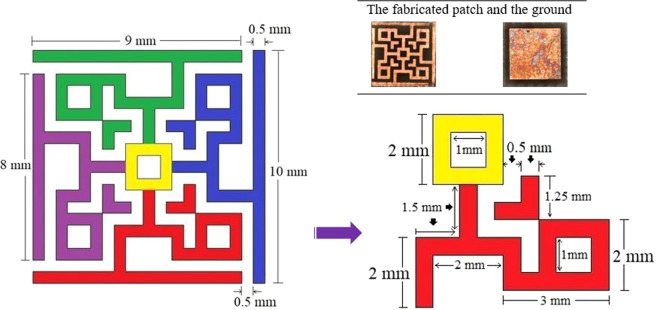
Dimension of the patch.

Usually, absorption with broad bandwidth and negative value of either permittivity or permeability or both, are the key objectives of designing an MM absorber. Any substrate can be used to design the patch on it with or without a ground at the opposite side of the patch. Commercially and widely used FR4 is not found much with broad bandwidth absorption in the X and Ku band regions. In this project, FR4 substrate (loss tangent = 0.025, dielectric constant = 4.3 and relative permeability = 1) with 1.578 mm thickness was used to design the proposed MM absorber unit cell. Fortunately, a broadband frequency absorption was achieved with so many attempts in the patch and the ground plane design with ending up to the following design shown in Fig. 1. The shape of the patch became like this from the intention of keeping square-shaped inductive and rectangular-shaped capacitive ring elements with 90-degree symmetric rotations. The metallic square rings are continuous for ensuring inductance and the rectangular rings are discontinuous by splits for capacitance. The entire unit cell is four-fold symmetrical as each quadrant are equivalent and rotated by 90 degrees. The size of the unit cell is 10 × 10 mm. The detailed dimension of the unit cell and a quadrant is shown in Fig. 2. Each quadrant is shown in different colors for the understanding of the patch design with the central square associated with all quadrants.

Each quadrant acts as an associate resonator to demonstrate the single negative value of either permittivity or permeability. The center of the total patch acts as a bridge to ensure equivocal current flow from any direction throughout the unit cell. The slits at the square border act as a capacitive load to perform for absorption in the lower frequencies (X band), whereas the discontinuities in the body of the patchwork for absorption in the higher frequencies (Ku band). Each quadrant with the center square and the perimeter lines act as inductive loads with complex values for reflection and transmission coefficients. The ground was chosen to reflect EM waves only to the quadrants except for the circumferential border transmission lines for better performance. This was because, the EM waves (any type of polarized) were needed to be rippled back from the ground to the patch so that the transmission coefficient of the unit cell is not negligible, which is essential to ensure negative values of permittivity and permeability.

The unit cell was fabricated (as shown in the top inset of Fig. 2) and data were taken using the cell in the PNA Network analyzer (N5227A).

### Metamaterial fundamentals and results

The absorption capability of an MM absorber can be calculated by

1 $$A(\omega )=1-R(\omega )-T(\omega )$$

if the reflection coefficient R (ω) and transmission coefficient T(ω) are zero, the highest absorption can be achieved. In the case of normal incidence of EM wave, the reflection coefficient can be achieved by

2 $$R(\omega )=\frac{Z(\omega )-{Z}_{o}}{Z(\omega )+{Z}_{o}}$$

where Z(ω) is the impedance of the MM absorber and z_o_ is the impedance of the free space (air). When Z(ω) = Z_o_ = 377 Ω, the reflection coefficient becomes zero. The characteristic or built-in impedance of the absorber can be found by

3 $$Z(\omega )=\sqrt{\frac{{(1+{S}_{11}(\omega ))}^{2}-{S}_{21}{(\omega )}^{2}}{{(1-{S}_{11}(\omega ))}^{2}-{S}_{21}{(\omega )}^{2}}}$$

as the designed unit cell has a symmetric patch, the dependence of characteristic impedance of the cell on the incident angle was not an issue. Furthermore, the groundsheet at the back of the substrate didn’t allow any EM waves to be transmitted through it. Hence the transmission coefficient was negligible and any amount of it was dissipated due to the dielectric loss tangent of the substrate medium. The simulated values of the reflection (S_11_) and transmission (S_21_) coefficients were found below −10dB at the resonance frequencies.

The refractive index (η) of the unit cell was calculated by two different methods: the Nicolson-Ross-Weir (NRW) method and the Direct Refractive Index (DRI) method as shown in the Eqs. (4) and (7) below.

4 $${\rm{By}}\,{\rm{NRW}}\,{\rm{method}},\,{\rm{\eta }}=-real[\sqrt{{\in }_{r}{\mu }_{r}}]$$

Where,

5 $${\in }_{r}({\rm{relative}}\,{\rm{permittivity}})=\frac{2}{\sqrt{-\frac{\omega }{c}d}}\frac{1-({S}_{21}+{S}_{11})}{1+({S}_{21}+{S}_{11})}$$

And

6 $${\mu }_{r}({\rm{relative}}\,{\rm{permeability}})=\frac{2}{\sqrt{-\frac{\omega }{c}d}}\frac{1-({S}_{21}-{S}_{11})}{1+({S}_{21}-{S}_{11})}$$

here *ω* = 2*πf* (*f* is the frequency of applied EM wave), d = thickness of the substrate and c = speed of light.

7 $${\rm{By}}\,{\rm{DRI}}\,{\rm{method}},\,{\rm{\eta }}={\rm{real}}\left[\frac{c}{i\pi fd}\sqrt{\frac{{({S}_{21}-1)}^{2}-{({S}_{11})}^{2}}{{({S}_{21}-1)}^{2}+{({S}_{11})}^{2}}}\right]$$

using Eq. (1) and (4) to (7), the results were found using simulated S_11_ and S_21_ parameters in transverse electric and magnetic (TEM) mode, which are tabulated in Table 1. The S parameters were found for 3 different polarized incident EM waves.

**Table 1:** Absorption and bandwidth of absorptions at different polarizing angles in TEM mode.

Polarizing Angle (normal incidence)	EM wave Mode	Frequency Band	Resonance Frequency (GHz)	Permittivity	Permeability	Refractive Index (NRW)	Refractive Index (DRI)	Max. Absorption	Absorption Bandwidth (GHz)
0-degree phi	TEM	X Band	11.31	**−0.9011**	2.095	**−1.023**	1.023	94.63%	**0.27**
	TEM	Ku Band	14.11	**−0.7755**	2.395	**−0.7518**	0.7518	95.58%	**0.87**
			14.23	1.905	**−0.4527**	**−0.6726**	0.6726	97%	
			14.69	2.728	**−0.1392**	**−0.6437**	0.6437	87.93%	
	TEM	Ku Band	17.79	6.37	**−0.2137**	**−0.9672**	0.9672	75.58%	**0.16**
			17.81	7.111	**−0.4196**	**−0.8144**	08144	73.8%	
45-degree phi	TEM	X Band	11.32	**−0.6305**	1.936	**−0.9998**	0.9998	95.47%	**0.26**
	TEM	Ku Band	14.13	**−0.3643**	1.99	**−0.7273**	0.7273	97.2%	**0.86**
			14.23	1.839	**−0.3765**	**−0.6722**	0.6722	97.12%	
			14.69	2.937	**−0.1533**	**−0.6562**	0.6562	86.7%	
	TEM	Ku Band	17.79	6.455	**−0.1749**	**−0.9875**	0.9875	75.29%	**0.14**
			17.81	7.183	**−0.3867**	**−0.8572**	0.8572	73.66%	
90-degree phi	TEM	X Band	11.32	**−0.6311**	1.937	**−0.9998**	0.9998	95.47%	**0.26**
	TEM	Ku Band	14.13	**−0.3642**	1.99	**−0.7273**	0.7273	97.2%	**0.86**
			14.21	1.493	**−0.1616**	**−0.6791**	0.6791	**98.15%**	
			14.69	2.931	**−0.1533**	**−0.6562**	0.6562	86.7%	
	TEM	Ku Band	17.79	6.455	**−0.1749**	**−1.018**	1.018	75.29%	**0.14**
			17.81	7.183	**−0.3867**	**−0.8572**	0.8572	**73.66%**	

**Figure 3:**
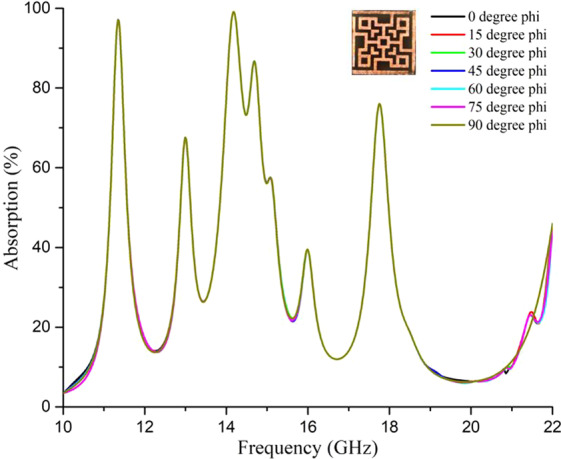
Absorption at normal incidence from simulation (TEM mode).

It is clear from Table 1 that, highest possible absorptions were found with a negative value of either permittivity or permeability. As a result, the refractive index became negative as per Eq. (5) & (6), which confirmed the metamaterial (MM) characteristics of the proposed unit cell. Also, the absorption bandwidth was calculated for frequencies with more than 70% absorption (for ensuring −10dB value of S_11_ and S_21_ parameters), which are quite good values and proved the design as a wide bandwidth MM absorber. The simulated absorption for plane-polarized EM waves at different normal incidences is plotted in Fig. 3, which shows similar performance with negligible distortions.

**Figure 4:**
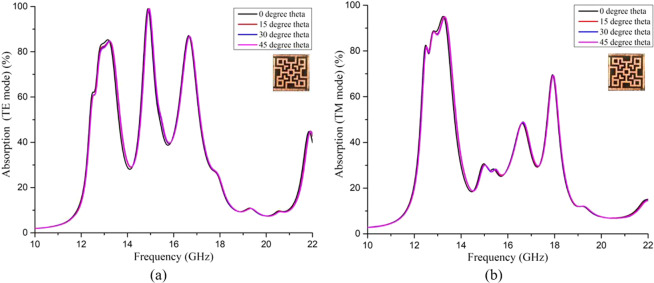
Absorption from simulation for oblique incidence at (**a**) TE mode and (**b**) TM mode operation.

The simulated absorptions for plane-polarized EM waves at different oblique incidences for TE (transverse electric) mode and TM (transverse magnetic) mode of applied incident waves are plotted in Fig. 4(a,b) respectively.

It is observed in Fig. 4(a) that, a considerable bandwidth of absorption (≈1.75 GHz) is ensured from the oblique incidence of applied EM waves on the proposed absorber in TE mode. Broadband absorption is achieved from 12.7 GHz to 13.46 GHz, 14.72 GHz to 15.19 GHz and 16.45 GHz to 16.97 GHz considering −10dB value of S_11_ and S_21_ parameters. Whereas in TM mode of applied EM wave at oblique incidence (Fig. 4(b)), the bandwidth of absorption reduces significantly to ≈1.24 GHz. It is important to mention that, in both TE and TM mode for oblique incidence, the proposed absorber can absorb only at Ku band, but bandwidth is increased compared to normal incidence.

**Figure 5:**
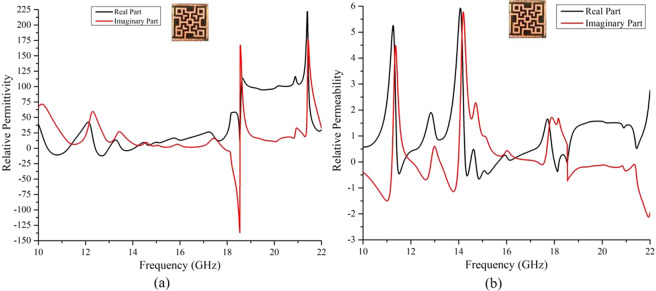
(**a**) Relative permittivity and (**b**) relative permeability of the absorber (from simulation) at operating frequencies.

At the resonance frequencies, the value of either relative permittivity or relative permeability was found negative as shown in Fig. 5, which ensures the negative value of the refractive index as per Eq. 4 & 7. Hence the absorption of applied EM waves at normal and oblique incidences is justified from the negative value of the refractive index.

**Figure 6:**
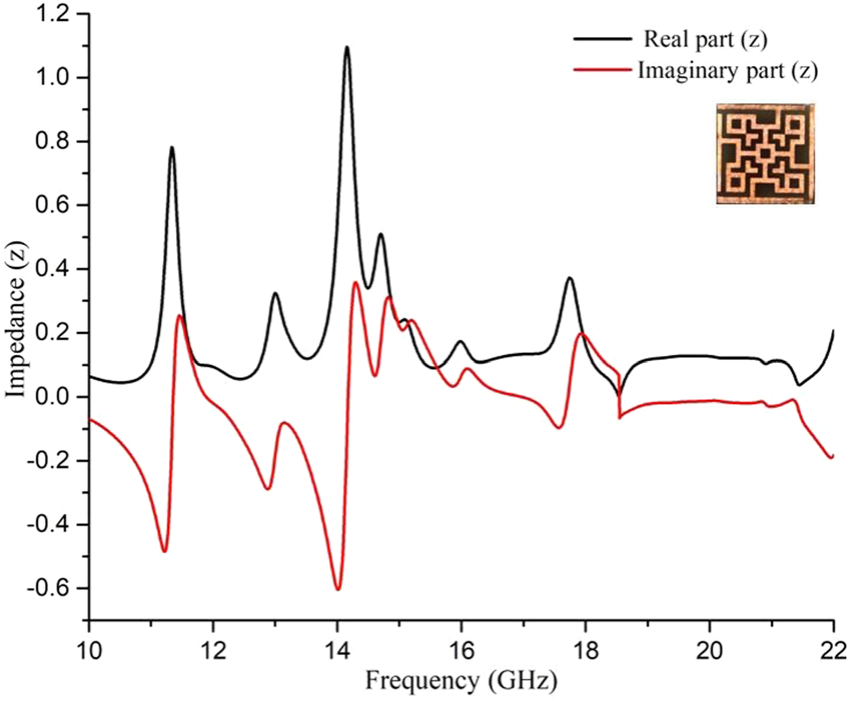
Impedance of the unit cell at operating frequencies.

The impedance of the unit cell in the whole operating frequencies is shown in Fig. 6. Impedance was determined by Eq. 3^20^.

**Figure 7:**
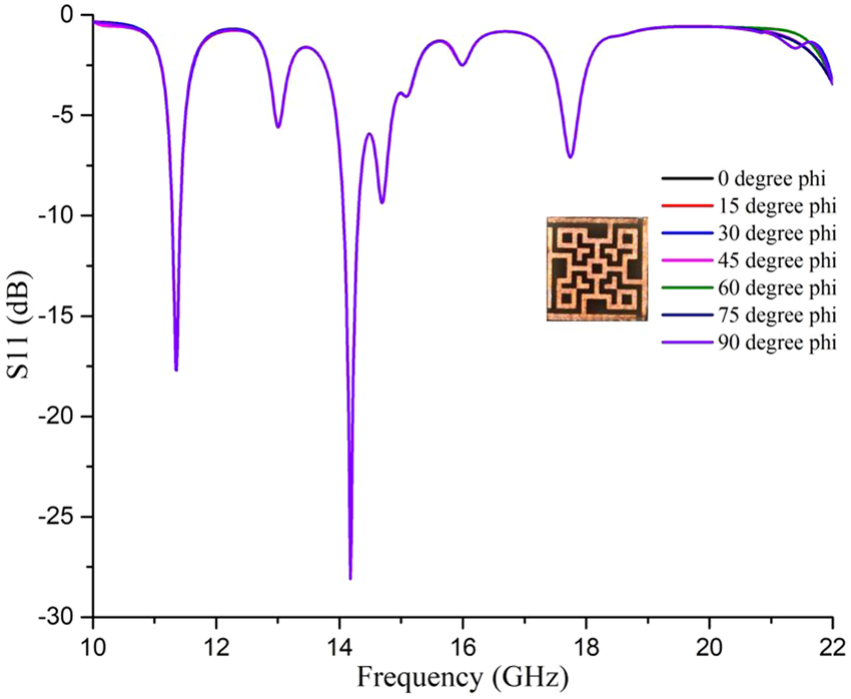
Reflection coefficient for normal incidences at operating frequencies.

From Fig. 6 it is clear that the impedance of the unit cell at the resonance frequencies aligns with the impedance of air, as a result, the reflection coefficient is less in these resonance frequencies as shown in Fig. 7. In association with transmission blocked by the metal ground, four absorption peaks were found at 11.31 GHz, 14.11 GHz, 14.23 GHz, and 17.79 GHz respectively.

**Figure 8:**
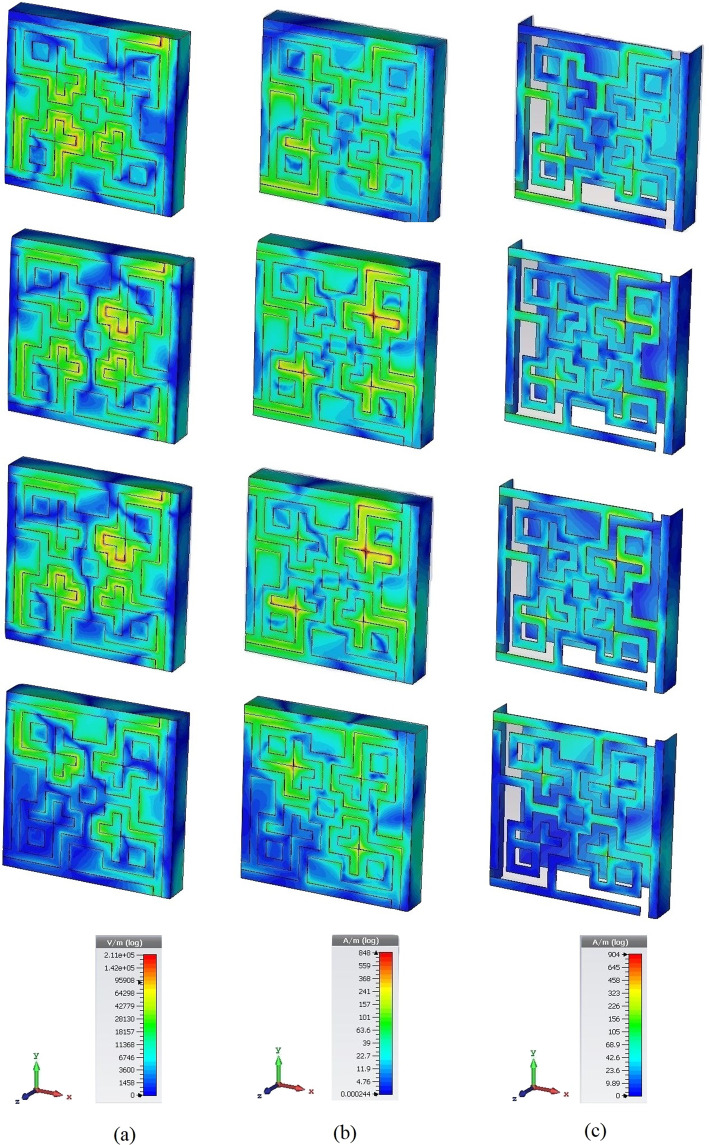
Instantaneous distribution of (**a**) electric field, (**b**) magnetic field, and (**c**) surface current at 11.31, 14.11, 14.23 and 17.79 GHz, respectively.

The behavior of the patch along with the ground at the resonance frequencies can be better understood by the following Fig. 8. The perfect electric field was applied along the x-axis, perfect magnetic field along the y-axis and the EM wave propagated along the z-axis. Hence *E*_*z*_ = 0 and *H*_*z*_ = 0 It is essential to mention that, the distributed fields and surface current are lying on the x-y plane. Hence the electric field has components *E*_*x*_ and *E*_*y*_. Similarly surface current and magnetic fields have *H*_*x*_, *H*_*y*_, and *J*_*x*_, *J*_*y*_ components respectively in Fig. 8. The electric field, magnetic field and current distributions at resonance frequencies show the respective dense amounts at some specific portions of the patch. The redder color shows the dense field and current density. Green color shows medium dense areas and blue means less dense. At the lowest resonance frequency (11.31 GHz), the lower-left corner of the patch seems to be agitated much than the upper right corner. And the right bottom and the upper left corners are comparatively less agitated. But in medium resonance frequencies (14.11 GHz and 14.23 GHz), all four corners are agitated. Hence highest absorption was found in these frequencies. Again, in the highest resonance frequency (17.79 GHz), all the corners except the lower-left corner are agitated. These happen because of the current flow throughout the patch transmission lines, which were changing directions with respect to frequencies. The distributions shown in Fig. 6 are polarization insensitive due to the symmetric geometry of the unit cell. In other words, the magnetic dipoles are aligned with the incident polarized magnetic field and hence the energy due to the incident magnetic field is trapped, which led to less reflection of EM waves and intense absorption inside the lossy dielectric material.

## Discussion

**Figure 9:**
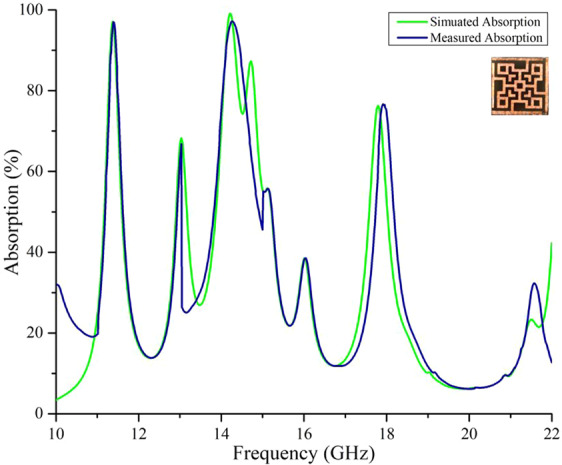
Simulated and measured absorption.

MM absorbers are designed to use for a perfect absorption of EM waves and other applications like sensing or imaging^21–23^. High-loss dielectric materials are needed for a wide bandwidth of absorption and curtailed thickness of the substrate. Whereas, for sensing or imaging applications, low-loss dielectric materials are appropriate^24^. For wideband absorption in the X and Ku band region, the proposed unit cell was designed. The simulated results were justified by taking measured data from VNA and were compared as shown in Fig. 9.

The simulated and measured data are quite similar except few deviations from resonance frequencies at the higher range. This is because the measured data were taken from two different waveguide ports (one port for 10–15 GHz and another port for 15–22 GHz). Absorption was calculated by Eq. 1, which needed S_11_ and S_21_ parameters. Hence for comparing the absorption by experimental data with simulated absorption, the total number of sample frequency points (from 10 GHz to 22 GHz) along the x-axis were taken equally. The changing of ports during measurement has caused some changes in expected values. Moreover, the high dielectric loss in the FR4 substrate at higher frequencies may be another reason and hence we see discrepancies in Fig. 9. The bandwidth of absorption is observed wide in Fig. 9. It is assumable that the proposed absorber can only absorb some fractions of the entire incident EM wave because the values of S_11_ and S_21_ parameters at the frequencies other than resonance have values more than −10 dB and hence there were absorptions less than 70%. As the substrate is a lossy material (FR4), it only has shown the necessary magnetic dipole momenta at the resonance frequencies.

The performance of the proposed absorber unit cell was compared with recent relevant works (by FR4 substrate) and found it more efficient in terms of response to wide-angle polarized TEM wave, maximum absorption and wide bandwidth of absorption along with the maximum number of resonance frequencies in X and Ku band as shown in Table 2.

**Table 2:** Comparison of proposed work with recent relevant works.

Reference	Year	Substrate Material	Copper patch only	Frequency Bands	Cell Structure Symmetry	Resonance Frequencies (GHz)	Unit Cell Size (mm×mm)	Polarization Insensitivity	Max Absorption (%)	The bandwidth of Absorbed Frequency (GHz)
[^25^]	2017	FR4	Yes	Ku	Yes	14.75, 15.1, 16.25	24 × 24	Up to 60°	95%	1.1
[^26^]	2017	FR4	Yes	X	Yes	8.08, 11.41	10 × 10	Up to 30°	98.97%	0.4
[^13^]	2017	FR4	Yes	X	Yes	10.1–10.6	17.6 × 17.6	Up to 70°	96.5%	0.35
[^27^]	2017	FR4	Yes	X	Yes	9.26	10 × 10	Up to 70°	95.28%	0.3
[^28^]	2018	FR4	No	X and Ku	Yes	9, 11, 13	15 × 15	Up to 65°	99%	Unknown
[^29^]	2019	FR4	Yes	C, X, and Ku	Yes	5.57, 7.96, 13.44	10 × 10	Up to 75°	99.28%	0.34
[^16^]	2019	FR4	Yes	X and Ku	Yes	8.6, 10.2, 11.95	10 × 10	Up to 60°	99.98%	1.05
Our Proposed Work	2019	FR4	Yes	X and Ku	Yes	11.21–11.49 13.92–14.85 17.66–17.87	10 × 10	Up to 90°	99.15%	1.42

## Methods

### Measurement

The unit cell was practically fabricated (as shown in Fig. 2) to test its absorbance from S parameters by Agilent PNA Network Analyzer N5227A with two different wave-guide ports (one is for 10–15 GHz and another one is for 15–22 GHz) connected simultaneously to the 2 ports of the VNA. In the simulation, the total number of points was 1021 (up to 15 GHz, 430 points and from 15.0119 GHz to 22 GHz, 591 points). To match experimental data with simulation, the number of points for the two waveguide ports were also set to 430 and 591 respectively on VNA and then combined to extract absorptions. As we have used two different waveguide ports, we had to calibrate the VNA setup for 10 to 15 GHz first for the first waveguide port for measurement and then calibrate again from 15 GHz to 22 GHz to measure using the second port. Calibration (e-cal) was done with Agilent N4694-60001 Electronic Calibration Module. S parameters (S_11_ and S_21_) were taken in real and imaginary parts separately and copied to an Excel file. Absorption was found from Matlab using data from the Excel file with the necessary codes. The measured values are quite similar to simulated values, which proves it a strong candidate for the absorption purpose in X and Ku band applications with features like the wide-incidence, angle, and polarization insensitivity and broad-bandwidth of frequencies absorptions.
